# Morphine Differentially Alters the Synaptic and Intrinsic Properties of D1R- and D2R-Expressing Medium Spiny Neurons in the Nucleus Accumbens

**DOI:** 10.3389/fnsyn.2019.00035

**Published:** 2019-12-20

**Authors:** Dillon S. McDevitt, Benjamin Jonik, Nicholas M. Graziane

**Affiliations:** ^1^Departments of Anesthesiology and Perioperative Medicine, and Pharmacology, Penn State College of Medicine, Hershey, PA, United States; ^2^Neuroscience Graduate Program, Penn State College of Medicine, Hershey, PA, United States; ^3^Medical Student Research Program, Penn State College of Medicine, Hershey, PA, United States

**Keywords:** nucleus accumbens, morphine, opioid use disorder, intrinsic excitability, synaptic transmission, neuronal activity

## Abstract

Exposure to opioids reshapes future reward and motivated behaviors partially by altering the functional output of medium spiny neurons (MSNs) in the nucleus accumbens shell. Here, we investigated how morphine, a highly addictive opioid, alters synaptic transmission and intrinsic excitability on dopamine D1-receptor (D1R) expressing and dopamine D2-receptor (D2R) expressing MSNs, the two main output neurons in the nucleus accumbens shell. Using whole-cell electrophysiology recordings, we show, that 24 h abstinence following repeated non-contingent administration of morphine (10 mg/kg, i.p.) in mice reduces the miniature excitatory postsynaptic current (mEPSC) frequency and miniature inhibitory postsynaptic current (mIPSC) frequency on D2R-MSNs, with concomitant increases in D2R-MSN intrinsic membrane excitability. We did not observe any changes in synaptic or intrinsic changes on D1R-MSNs. Last, in an attempt to determine the integrated effect of the synaptic and intrinsic alterations on the overall functional output of D2R-MSNs, we measured the input-output efficacy by measuring synaptically-driven action potential firing. We found that both D1R-MSN and D2R-MSN output was unchanged following morphine treatment.

## Introduction

Exposure to opioids reshapes future reward and motivated behaviors partially by altering the functional output of medium spiny neurons (MSNs) in the nucleus accumbens shell, a brain region central to reward and motivation (Wolf, 2010; Graziane et al., 2016; Hearing et al., 2016; Scofield et al., 2016). MSNs receive glutamatergic excitatory input from the infralimbic prefrontal cortex, amygdala, hippocampus, and midline nuclei of the thalamus, while also receiving inhibitory input locally from interneurons or collateral projections from MSNs or from other brain regions including the ventral pallidum, lateral septum, periaqueductal gray, parabrachial nucleus, pedunculopontine tegmentum and ventral tegmental area (Sesack and Grace, 2010; Lalchandani et al., 2013; Salgado and Kaplitt, 2015; Dobbs et al., 2016; McDevitt and Graziane, 2018). The integration of these synaptic inputs along with the intrinsic excitably of MSNs are, in part, critically important for information transfer through the reward neurocircuit (Russo et al., 2010; Kourrich et al., 2015).

There are two main classes of MSNs in the accumbens shell; dopamine D1-receptor containing and dopamine D2-receptor containing MSNs (D1R-MSN and D2R-MSN, respectively). These cell-types not only differ in the dopamine receptor expressed, but also in their projection sites, peptidergic expression, and modulation of motivated behaviors (Hikida et al., 2010; Lobo et al., 2010; Smith et al., 2013; Koo et al., 2014; Al-Hasani et al., 2015; Creed et al., 2016; Heinsbroek et al., 2017; Tejeda et al., 2017; Castro and Bruchas, 2019). Recently, reports have demonstrated that exposure to morphine differentially alters excitatory glutamatergic transmission on both D1R- and D2R-MSNs in the accumbens shell (Graziane et al., 2016; Hearing et al., 2016, 2018; Madayag et al., 2019). However, little is known regarding how exposure to morphine alters MSN cell-type-specific inhibitory transmission and intrinsic membrane excitability, or how these synaptic and intrinsic factors integrate to drive future D1R- or D2R-MSN functional output. In an attempt to identify the effect of morphine exposure on D1R- and D2R-MSN functional output in the accumbens shell, we investigated how repeated exposure to morphine affected the integration of excitatory and inhibitory transmission, along with the intrinsic factors that drive membrane excitability. Finally, we assessed the integrated effect that synaptic and intrinsic factors had on the overall functional output of MSNs in the accumbens 24 h following morphine administration.

## Materials and Methods

### Animals

All experiments were done in accordance with procedures approved by the Pennsylvania State University College of Medicine Institutional Animal Care and Use Committee. Cell-type-specific D1R- or D2R-MSN recordings were made using male and female B6 *Cg-Tg* (*Drd1a*-tdTomato) line 6 Calak/J hemizygous mice, a bacterial artificial chromosome (BAC) transgenic mouse line initially developed in the laboratory of Dr. Nicole Calakos at Duke University, aged 5–10 weeks (Ade et al., 2011; JAX stock #16204). Given that in this transgenic mouse line, D1R-MSNs are fluorescently labeled, D2R-MSNs were identified based on the lack of fluorescence, cell size, and electrophysiological characteristics, including capacitance and membrane resistance (Table 1), as previously published (Graziane et al., 2016). Additionally, as elegantly stated previously (Willett et al., 2019), unlabeled MSNs in the *Drd1a*-tdTomato line in adult mice nearly exclusively compromise *Drd2*-positive MSNs and to a lesser extent MSNs expressing both D1R and D2R (D1R/D2R-MSNs; 1.6%; Ade et al., 2011; Enoksson et al., 2012; Thibault et al., 2013). Thus, we refer to all unlabeled MSNs from the *Drd1a*-tdTomato line as D2R-MSNs, but with the full acknowledgment that we are also likely sampling from D1R/D2R-MSNs, but to a much lesser degree (Bertran-Gonzalez et al., 2008; Ade et al., 2011). Mice were singly-housed and maintained on a regular 12 h light/dark cycle (lights on 07:00, lights off 19:00) with *ad libitum* food and water.

**Table 1:** Cell Properties from electrophysiological assessments.

	Saline		Morphine	
	D1R-MSN	D2R-MSN	D1R-MSN	D2R-MSN
Membrane Capacitance (pF)	81.11 ± 2.11 (71)	75.83 ± 2.56 (64)	82.01 ± 2.48 (72)	76.93 ± 1.89 (78)
Membrane Resistance (MΩ)	250.5 ± 9.09 (71)	256.1 ± 12.34 (64)	246.6 ± 11.88 (72)	280.3 ± 10.8 (78)
Tau (ms)	1.53 ± 0.07 (21)	1.42 ± 0.09 (16)	1.49 ± 0.08 (21)	1.49 ± 0.12 (16)

*Number of cells (n). Tau values provided by Axon software. They were unavailable in Sutter Software, which results in a lower n*.

### Drugs

(−)-morphine sulfate pentahydrate was provided by the National Institute on Drug Abuse Drug Supply Program. NBQX and AP5 were purchased from Tocris Biosciences. Picrotoxin was purchased from Sigma Aldrich. Tetrodotoxin (TTX) was purchased from Enzo.

### Repeated Systemic Injections of Saline or Morphine

Before drug administration, mice were allowed to acclimate to their home cages for >5 days. For drug treatment, we used a 5 day repeated drug administration procedure (Huang et al., 2009; Graziane et al., 2016). In all electrophysiological experiments, once per day for 5 days, mice were taken out of the home cages for an intraperitoneal (i.p.) injection of either (−)-morphine sulfate pentahydrate (10 mg/kg in 0.9% saline) or the same volume of 0.9% saline, and then placed back to the home cage at ~Zeitgeber time (ZT) 2 (ZT0 = lights on, ZT12 = lights off). Animals were randomly selected for each drug treatment. Morphine- or saline-treated animals were then used for electrophysiological recordings ~24 h following the last injection.

### Acute Brain Slice Preparation

At ~ZT time 2, mice were deeply anesthetized with isoflurane and cardiac perfused with an ice-cold NMDG-based cutting solution containing (in mM): 135 N-methyl-d-glucamine, 1 KCl, 1.2 KH_2_PO_4_, 0.5 CaCl_2_, 1.5 MgCl_2_, 20 choline-HCO_3_, and 11 glucose, saturated with 95%O_2_/5%CO_2_, adjusted to a pH of 7.4 with HCl, osmolality adjusted to 305 mmol/kg. Following perfusion, mice were decapitated and brains were rapidly removed. Two-hundred and fifty micrometer coronal brain slices containing the nucleus accumbens shell were prepared *via* a Leica VT1200s vibratome in 4°C NMDG cutting solution. Following cutting, slices were allowed to recover in artificial cerebrospinal fluid (aCSF) containing (in mM): 119 NaCl, 2.5 KCl, 2.5 CaCl_2_, 1.3 MgCl_2_, 1 NaH_2_PO_4_, 26.2 NaHCO_3_, and 11 glucose, osmolality of 290 mmol/kg, at 31°C for 30 min followed by 30 min at 20–22°C prior to recording. After a 1 h recovery period, slices were kept at 20–22°C for the rest of the recording day.

### Electrophysiology

Whole-cell recording. All recordings were made from the nucleus accumbens shell between Bregma 1.7 mm and 0.86 mm (Paxinos and Franklin, 2004). Slices were transferred to a recording chamber and neurons were visualized using infrared differential interference contrast microscopy. During recording, slices were superfused with aCSF at room temperature. For intrinsic membrane excitability experiments, recording electrodes [2–5 MΩ; borosilicate glass capillaries (WPI #1B150F-4) pulled on a horizontal puller from Sutter Instruments (model P-97)] were filled with a potassium-based internal solution containing (in mM): 130 KMeSO_3_, 10 KCl, 10 HEPES, 0.4 EGTA, 2 MgCl_2–_6H_2_0, 3 Mg-ATP, 0.5 Na-GTP, pH 7.2–7.4, osmolality = 290 mmol/kg (Wescor Vapro Model 5,600, ElitechGroup). Resting membrane potential was recorded immediately following break-in. Before beginning the protocol, cells were adjusted to a resting membrane voltage of −80 mV. This typically was achieved with less than 30 pA current injection, and cells were discarded if the current needed to adjust the cell to −80 mV was greater than 50 pA. A current step protocol consisting of 600 ms steps ranging from −200 to +450 pA in 50 pA increments was carried out with a 20 s intra-sweep interval. The number of action potentials observed at each current step was recorded.

For synaptically-driven action potential experiments or rheobase/chronaxie measurements, a stimulation electrode (size, 2.5–3 MΩ), filled with aCSF, was placed 100 μm from the recorded neuron along the same z plane in three-dimensional space. Recordings were performed using KMeSO_3_ as described above. The resting membrane potential was not adjusted, enabling neurons to fire action potentials. The average membrane potential during electrophysiology recordings was −85.3 ± 0.78 mV, which deviated by 4.39 ± 0.40 mV (*n* = 50) throughout the entirety of the experiment. For synaptically-driven action potential experiments, a 10 Hz stimulus with a stimulus duration of 0.25 ms and stimulus strength ranging from 0 to 100 μAmps of current, with an interval of 5 μAmps, was applied through the stimulating electrode. For each current, this procedure was repeated three times and the average number of action potentials/10 Hz stimulus was recorded. Rheobase/chronaxie measurements were made by varying the stimulus duration from 2 to 0.2 μAmps and injecting current at each duration until an action potential was evoked from the recorded neuron. The stimulus duration was plotted over the current which elicited an action potential. The rheobase was calculated as the plateau of a two-phase decay nonlinear regression curve fit. The chronaxie was calculated, using GraphPad Prism software, as the duration corresponding to 2× the rheobase, by solving for x in the equation, rheobase*2 = rheobase + SpanFast*exp (−KFast*x) + SpanSlow*exp (−KSlow*x).

For excitatory/inhibitory ratio (E/I) experiments (Liu et al., 2016), recording electrodes (2–5 MΩ) were filled with a cesium-based internal solution (in mM): 135 CsMeSO3, 5 CsCl, 5 TEA-Cl, 0.4 EGTA (Cs), 20 HEPES, 2.5 Mg-ATP, 0.25 Na-GTP, 1 QX-314 (Br), pH 7.2–7.4, osmolality = 290 mmol/kg. This internal solution was selected (i) to isolate synaptically-evoked currents (cesium and QX-314 block voltage-gated K^+^ and Na^+^ channels, respectively); and (ii) to measure the E/I ratios at physiologically relevant ionic driving forces while MSNs were voltage-clamped at −70 mV (−70 mV is similar to the membrane potential of MSNs during synaptically-driven action potential and rheobase/chronaxie measurements, which were performed in current-clamp; using this internal solution the reversal potential for γ-aminobutyric acid_A_ (GABA_A_) receptor/glycine receptors (receptors likely mediating inhibitory postsynaptic currents (IPSCs) and AMPA/kainate receptors (receptors mediating excitatory postsynaptic currents, EPSCs) is ~−60 mV and ~0 mV, respectively). To evoke postsynaptic currents, presynaptic afferents were stimulated *via* a constant-current stimulator (Digitimer) using a monopolar stimulating electrode (glass pipette filled with aCSF) at 0.1 Hz with 0.1 ms stimulus duration. Cells were held at −70 mV for the entirety of the experiment. Once a stable baseline was observed near 200 pA of current, 50 traces were recorded. Following this, NBQX (2 μM) and AP5 (50 μM) were both applied to isolate inhibitory ionotropic receptor-mediated currents. The drug was allowed to wash on, and 50 more sweeps were recorded. The AMPA/kainate receptor-mediated current was then obtained *via* digital subtraction of the inhibitory ionotropic receptor-mediated current from the mixed current. The E/I ratio was then calculated by taking the peak amplitude of the AMPA/kainate receptor-mediated current divided by the peak amplitude of the inhibitory ionotropic receptor-mediated current in male or female mice.

E-I balance assessments investigating temporal relationships between excitatory and inhibitory current were carried out in male mice by measuring spontaneous events using cesium based internal solution (see recipe above) and aCSF. Neurons were held at −30 mV in order to elicit inward excitatory current and outward inhibitory current, as done previously (Zhou et al., 2009). Recordings lasted 3 min and analysis was performed using MiniAnalysis software. A computer program built in Visual Studio was used to calculate the inter-event intervals of sEPSC and sIPSCs.

Miniature excitatory or inhibitory postsynaptic current (mEPSC or miniature inhibitory postsynaptic current, mIPSC, respectively) recordings were performed in the presence of tetrodotoxin (1 μM), a Na^+^ channel blocker. mEPSCs were recorded in the presence of picrotoxin (100 μM) and mIPSCs were recorded in the presence of NBQX (2 μM). For mEPSC recordings, recording electrodes (2–5 MΩ) were filled with the cesium-based internal solution as described above. For mIPSC recordings, recording electrodes (2–5 MΩ) were filled with high chloride cesium-based internal solution (in mm): 15 CsMeSO3, 120 CsCl, 8 NaCl, 0.5 EGTA (Cs), 10 HEPES, 2.0 Mg-ATP, 0.3 Na-GTP, 5 QX-314 (Br), pH 7.2–7.4, osmolality = 290 mmol/kg. High chloride cesium-based internal solution was used for mIPSC recordings so that mIPSCs could be detected in neurons voltage clamped at −70 mV (γ-aminobutyric acid_A_ (GABA_A_) receptor/glycine receptor reversal potential = ~0 mV). Events during a stable 10 min period were analyzed using Sutter software Pernía-Andrade et al., 2012). Decay tau corresponds to the time constant of decay time, which equals the 10–90% decay time. The rise time equals 10–90% rise time.

All recordings were performed using either an Axon Multiclamp 700B amplifier or Sutter Double IPA, filtered at 2–3 kHz, and digitized at 20 kHz. Series resistance was typically 10–25 MΩ, left uncompensated, and monitored throughout. For all voltage-clamp recordings, cells with a series of resistance variation greater than 20% were discarded from the analysis. For all current-clamp recordings, cells with a bridge balance that varied greater than 20% during the start and end of recordings were discarded from analysis.

### Statistical Analysis

All results are shown as mean ± SEM. Each experiment was replicated in at least three animals. No data points were excluded. The sample size was presented as n/m, where “n” refers to the number of cells and “m” refers to the number of animals. Statistical significance was assessed in GraphPad Prism software using a one- or two-way ANOVA with Bonferroni’s correction for multiple comparisons in order to identify differences as specified. *F*-values for two-way ANOVA statistical comparisons represent interactions between variables unless otherwise stated. Two-tail tests were performed for all studies. Our goal, *a priori*, was to examine pairwise comparisons between drug treatment and cell type combinations regardless if the interaction effect between drug treatment and cell type was strong. Thus, prior to analysis, we created all possible independent groups based on drug treatment and cell type combinations and performed a one-way ANOVA with pairwise comparisons. The results from these pairwise comparisons from this one-way ANOVA would be equivalent to performing a two-way ANOVA with an interaction term (drug treatment, cell type, drug treatment*cell type interaction) and then performing *post hoc* pairwise comparisons on the interaction term from the two-way ANOVA model.

## Results

### Morphine Reduces Synaptic Transmission on D2R-MSNs

**Figure 1:**
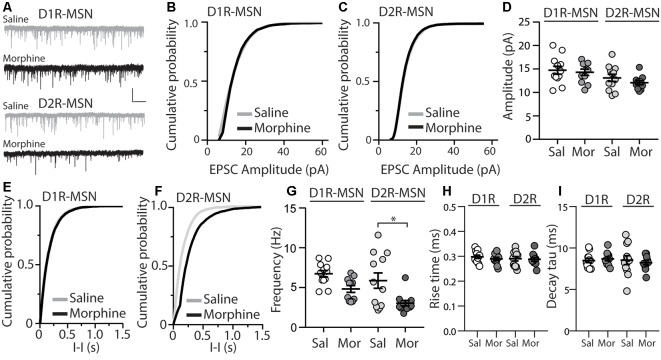
Repeated morphine administration reduces miniature excitatory postsynaptic current (mEPSC) frequency on dopamine D2-receptor medium spiny neurons (D2R-MSNs) on abstinence day 1. **(A)** Representative traces showing mEPSCs recorded from D1R- or D2R-MSNs from animals treated with saline or morphine (10 mg/kg, i.p.). Scale bar: 20 pA, 1 s. **(B,C)** Cumulative plot of a representative neuron showing the distribution of mEPSC amplitudes recorded from D1R-MSNs **(B)** or D2R-MSNs **(C)** in animals treated with saline or morphine. **(D)** Summary graph showing the average mEPSC amplitude recorded D1R- or D2R-MSNs following saline or morphine treatment (*F*_(3,42)_ = 2.92, *p* = 0.045; One-way ANOVA. Bonferroni post-test, D1R-MSN: saline vs. morphine, *p* > 0.999; D2R-MSN: saline vs. morphine, *p* > 0.999). **(E,F)** Cumulative plot of a representative neuron showing the distribution of mEPSC inter-event intervals (I-I) recorded from D1R-MSNs **(E)** or D2R-MSNs **(F)** in animals treated with saline or morphine. **(G)** Summary graph showing the average mEPSC frequency recorded from D1R- or D2R-MSNs following saline or morphine treatment (*F*_(3,42)_ = 6.73, *p* = 0.0008; One-way ANOVA with Bonferroni post-test; *p < 0.05). **(H)** Summary graph showing the average rise time of mEPSC recorded from D1R- or D2R-MSNs following saline or morphine treatment. **(I)** Summary graph showing the average decay tau of mEPSC recorded from D1R- or D2R-MSNs following saline or morphine treatment. Circle = neuron.

Previously, it was found that exposing mice to a dosing regimen (i.p. 10 mg/kg per day for 5 days, 1-day forced abstinence) that induces locomotor sensitization and conditioned place preference generates silent synapse expression preferentially on D2R-MSNs, but not D1R-MSNs, *via* removal of AMPA receptors from mature synapses (Graziane et al., 2016). The removal of AMPA receptors from the synapse is expected to change the number of release sites (n) when AMPA receptor-mediated transmission is the readout (Hanse et al., 2013), which results in a change in frequency of quantal events (Kerchner and Nicoll, 2008). Based on this, we assessed morphine-induced quantal changes in D1R- or D2R-MSN synaptic transmission by measuring mEPSCs. We found that 24 h following repeated morphine treatment, D1R-MSNs showed no changes in mEPSC amplitude (Bonferroni post-test, *p* > 0.999; Figures 1A,B,D), and this was also observed on D2R-MSNs (Bonferroni post-test, *p* > 0.999; Figures 1A,C,D). Furthermore, analysis of mEPSC frequency following morphine exposure showed no effect on D1R-MSNs (Bonferroni post-test, *p* = 0.19; Figures 1E,G). However, *post hoc* analysis revealed a significant difference between mEPSC frequency following morphine exposure on D2R-MSNs (Bonferroni post-test, *p* = 0.01; Figures 1F,G). Last, we analyzed the receptor rise time and decay tau of mEPSCs, in order to measure whether the significant effects observed, were potentially mediated by changes in AMPA/kainate receptor kinetics. We found, in all groups, the receptor kinetics, rise time and decay tau, remained unchanged (Rise time: *F*_(3,42)_ = 0.371, *p* = 0.77; One-way ANOVA; decay tau: *F*_(3,42)_ = 0.290, *p* = 0.83; One-way ANOVA; Figures 1H,I). Based on previously published findings (Graziane et al., 2016), it is likely that the observed morphine-induced decreases in D2R-MSN mEPSC frequency are caused by a reduction in the number of release sites (n) due to morphine-induced AMPA receptor removal from mature D2R-MSN synapses.

**Figure 2:**
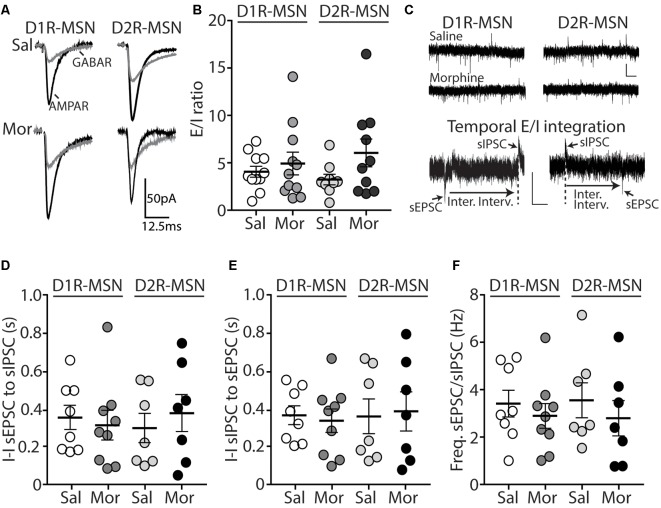
Short-term abstinence from *in vivo* morphine treatment has no effect on the evoked excitatory/inhibitory (E/I) ratio and does not alter the temporal relationship between spontaneous EPSCs (sEPSCs) and spontaneous inhibitory postsynaptic currents (sIPSCs) on D1R- or D2R-MSNs in the nucleus accumbens shell. **(A)** Representative traces showing evoked AMPA receptor (AMPAR)- and GABA receptor (GABAR)-mediated currents on D1R- or D2R-MSNs 24 h following repeated saline or morphine treatments. Neurons were held at −70 mV. **(B)** Summary graph showing the E/I ratio of evoked currents on D1R- or D2R-MSNs 24 h following repeated saline (sal) or morphine (mor; 10 mg/kg, i.p.) treatments. There were no significant differences between groups in male or female mice. **(C)** Representative traces showing spontaneous EPSCs (inward current) and IPSCs (outward current) when D1R- or D2R MSNs were held at −30 mV 24 h following *in vivo* morphine treatment. Scale bars: 20 pA, 0.5 s (Lower). Electrophysiological recordings in whole-cell patch-clamp configuration showing the inter-event intervals (inter. interv.) of sEPSCs to sIPSCs (left) or sIPSCs to sEPSCs (right) in a MSN held at −30 mV. Scale bars: 20 pA, 0.125 s. **(D)** Summary graph showing no significant changes in the inter-event interval (I-I) between sEPSCs and sIPSCs on D1R- or D2R-MSNs 24 following repeated saline or morphine administration in male mice. **(E)** Summary graph showing no significant changes in the inter-event interval (I-I) between sIPSCs and sEPSCs on D1R- or D2R-MSNs 24 following repeated saline or morphine administration in male mice. **(F)** Summary graph showing that morphine exposure had no effect on the frequency ratio of sEPSC to sIPSC events within D1R- or D2R-MSNs in male mice.

The functional output of MSNs in the nucleus accumbens relies upon the integration of excitatory and inhibitory synaptic transmission (Plenz and Kitai, 1998; Wickens and Wilson, 1998; Wolf et al., 2005; Otaka et al., 2013). To measure whether morphine-induced changes in mEPSC frequency on D2R-MSNs are sufficient to impact the excitatory and inhibitory balance of synaptic input, we measured the ratio of excitatory ionotropic receptor-mediated current to inhibitory ionotropic receptor-mediated current (E/I ratio) following an electrically evoked stimulus while MSNs were voltage-clamped at −70 mV. We found that 24 h post morphine treatment the E/I ratios were unchanged on D1R- or D2R-MSNs (*F*_(3,37)_ = 1.27, *p* = 0.30; One-way ANOVA; Figures 2A,B). Given that the temporal integration of excitatory and inhibitory synaptic transmission regulates neuronal activity (Wehr and Zador, 2003; Higley and Contreras, 2006; Okun and Lampl, 2008; Hiratani and Fukai, 2017; Roland et al., 2017; Bhatia et al., 2019), we investigated whether morphine administration altered the temporal relationship between excitation and inhibition on D1R- or D2R-MSNs. In order to test this, we recorded spontaneous EPSCs (sEPSCs) and sIPSCs while voltage clamping D1R- or D2R-MSNs at −30 mV, which enabled us to simultaneously detect EPSCs (inward current with a reversal at ~0 mV, Lee et al., 2013) and IPSCs (outward current with a reversal potential at ~−60 mV; Table 2) within each neuron, as previously demonstrated (Zhou et al., 2009). Measuring spontaneous activity was chosen in order to sample both action potential mediated and non-action potential mediated events, encompassing synaptic populations sampled during evoked stimulation or miniature postsynaptic current recordings, respectively (He et al., 2018). With this approach, we were able to measure the temporal relationship between sEPSCs and sIPSCs as well as the balance of excitatory to inhibitory transmission on D1R- or D2R-MSNs (Figure 2C). Our results show that morphine exposure did not alter the temporal relationship between excitatory and inhibitory events as we did not observe any changes in the inter-event interval between sEPSCs to sIPSCs (*F*_(3,27)_ = 0.198, *p* = 0.90, one-way ANOVA; Figure 2D) or from sIPSCs to sEPSCs (*F*_(3,27)_ = 0.072, *p* = 0.97, one-way ANOVA; Figure 2E). Additionally, we did not observe any morphine-induced changes in the excitation to inhibition balance measured by taking the sEPSC/sIPSC frequency ratio on D1R- or D2R-MSNs (*F*_(3,27)_ = 0.339, *p* = 0.80, one-way ANOVA; Figure 2F), suggesting that the relationship between spontaneous postsynaptic excitatory and inhibitory currents within a neuron are unaffected by morphine treatment, despite the observed changes in mEPSC frequency.

**Table 2:** Calculated Cl^−^ reversal potential for D1R- or D2R-MSNs in the nucleus accumbens shell.

Saline		Morphine	
D1R-MSN	D2R-MSN	D1R-MSN	D2R-MSN
−59.09 ± 1.2 mV; *n* = 10/7	−60.98 ± 1.5 mV; *n* = 8/5	−59.19 ± 1.7 mV; *n* = 5/4	−60.94 ± 1.0 mV; *n* = 4/4

*Cl^−^ reversal potential was calculated in the whole-cell patch-clamp configuration using cesium methanesulfonate internal solution with bath application of aCSF+NBQX (2 μM) and AP5 (50 μM). Whole-cell patch-clamp configuration was used to mimic the approach used in spontaneous EPSC and IPSC recordings (Figure 2). The values were corrected with a junction potential of 10.4 mV (V_m_ = V_p_ − V_L_ where V_m_ = the membrane voltage, V_p_ = the calculated voltage, and V_L_ is the voltage of the liquid junction potential; Figl et al., 2003). N/m = number of cells/number of animals*.

**Figure 3:**
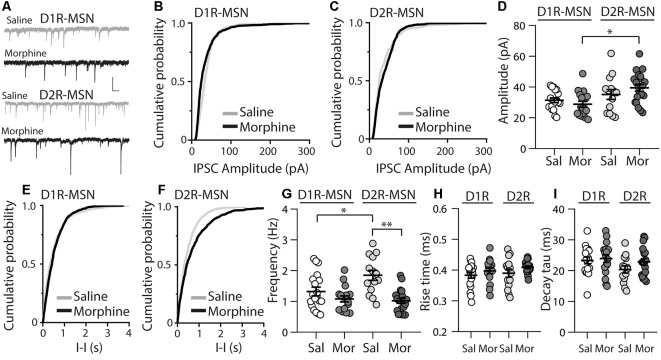
Repeated morphine administration reduces miniature inhibitory postsynaptic current (mIPSC) frequency on D2R-MSNs on abstinence day 1. **(A)** Representative traces showing mIPSCs recorded from D1R- or D2R-MSNs from animals treated with saline or morphine (10 mg/kg, i.p.). Scale bar: 25 pA, 0.5 s. **(B,C)** Cumulative plot showing the distribution of mIPSC amplitudes recorded from D1R-MSNs **(B)** or D2R-MSNs **(C)** in animals treated with saline or morphine. **(D)** Summary graph showing the average mIPSC amplitude recorded D1R- or D2R-MSNs following saline or morphine treatment (*F*_(3,65)_ = 4.73, *p* = 0.005; one way ANOVA with Bonferroni post-test). *p < 0.05. **(E,F)** Cumulative plot of a representative neuron showing the distribution of mIPSC inter-event intervals (I-I) recorded from D1R-MSNs **(E)** or D2R-MSNs **(F)** in animals treated with saline or morphine. **(G)** Summary graph showing the average mIPSC frequency recorded from D1R- or D2R-MSNs following saline or morphine treatment (*F*_(3,65)_ = 8.94, *p* < 0.0001; one-way ANOVA with Bonferroni post-test; *p < 0.05, **p < 0.01). **(H)** Summary graph showing the average rise time of mIPSC recorded from D1R- or D2R-MSNs following saline or morphine treatment. **(I)** Summary graph showing the average decay tau of mIPSC recorded from D1R- or D2R-MSNs following saline or morphine treatment. Circle = neuron.

Because E/I ratios are dependent upon changes in excitatory and/or inhibitory synaptic transmission, we next investigated whether inhibitory transmission on D1R- or D2R-MSNs was altered 24 h following morphine treatment, by measuring miniature inhibitory postsynaptic currents (mIPSCs; Figure 3A). First, we measured the mIPSC amplitude on D1R- or D2R-MSNs. We found that following morphine treatment, there was no significant change in the mIPSC amplitude on D1R-MSNs (Bonferroni post-test, *p* > 0.999; Figures 3B,D) or on D2R-MSNs (Bonferroni post-test, *p* > 0.999; Figures 3C,D). Furthermore, when measuring the mIPSC frequency, our data revealed no significant morphine-induced change on D1R-MSNs (Bonferroni post-test, *p* = 0.949; Figures 3E,G). However, morphine abstinence elicited a significant decrease in mIPSC frequency on D2R-MSNs (Bonferroni post-test, *p* < 0.0001; Figures 3F,G). We also found that basal levels of mIPSC frequency were significantly greater on D2R-MSNs compared to D1R-MSNs (Bonferroni post-test, *p* = 0.02). Lastly, to measure whether inhibitory ionotropic receptor kinetics was potentially a factor in the observed changes, we measured mIPSC rise time and decay tau (Figures 3H,I). We found, in all groups, the receptor kinetics, rise time and decay tau, remained unchanged (rise time: *F*_(3,65)_ = 1.69, *p* = 0.18; decay tau: *F*_(3,65)_ = 1.62, *p* = 0.19; one-way ANOVA).

### Morphine Increases the Intrinsic Membrane Excitability of D2R-MSNs

MSNs in the nucleus accumbens shell display bistable membrane potential properties characterized by a hyperpolarized quiescent “down” state and a depolarized “up” state associated with neuronal discharge (O’Donnell et al., 1999). These states are controlled by combined excitatory synaptic discharge and intrinsic membrane excitability (Huang et al., 2011), which are posited to bring the membrane potential close to the MSN firing threshold, thus impacting the efficiency of information relay to downstream brain regions (O’Donnell and Grace, 1995; Ishikawa et al., 2009). Given our observed changes in synaptically-mediated excitatory and inhibitory transmission on D2R-MSNs (Figures 1, 3), our next experiment tested whether morphine impacts cell-type specific MSN intrinsic membrane excitability. To do this, using whole-cell electrophysiological recordings, we measured the number of action potentials in response to depolarizing currents, as this approach is often used to measure intrinsic membrane excitability (Desai et al., 1999; Nelson et al., 2003; Zhang and Linden, 2003; Heng et al., 2008; Ishikawa et al., 2009; Wang et al., 2018). We found that during morphine abstinence, there were no changes on D1R-MSN membrane excitability (Bonferroni post-test at each current injected, *p* > 0.999; 4A,B). However, the morphine-induced decreases in synaptic input onto D2R-MSNs (Figures 1, 3) were accompanied by an overall increase in the intrinsic membrane excitability at current injections of ≥250 pA (Bonferroni post-test, 250 pA: *p* = 0.008; 300 pA: *p* = 0.0003; 350–450 pA: *p* < 0.0001; 4A,C).

**Figure 4:**
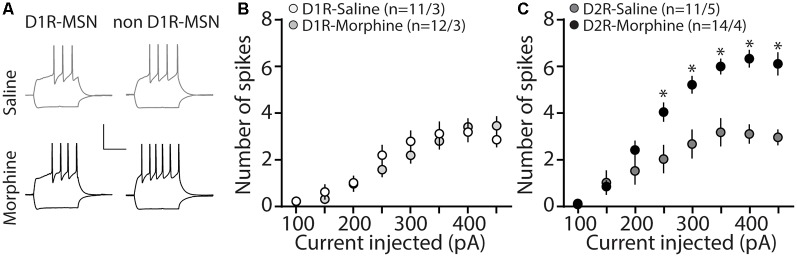
Repeated morphine administration increases membrane excitability on D2R-MSNs on abstinence day 1. **(A)** Representative traces, scale bar, 40 mV, 300 ms at 100 pA current injection. **(B)** Summary graph showing the average number spikes generated by injected current on D1R-MSNs following saline or morphine (10 mg/kg, i.p.) treatment (*F*_(7,238)_ = 1.05, *p* = 0.395; two-way repeated-measures ANOVA). **(C)** Summary graph showing the average number of spikes generated by the injected current on D2R-MSNs following saline or morphine treatment (*F*_(7,210)_ = 10.4, *p* = *0* < 0.0001; two-way repeated-measures ANOVA with Bonferroni post-test). *p < 0.05. (n/n = cells/animals).

### D2R-MSN Synaptically Driven Functional Output Is Unchanged Following Morphine Treatment

**Figure 5:**
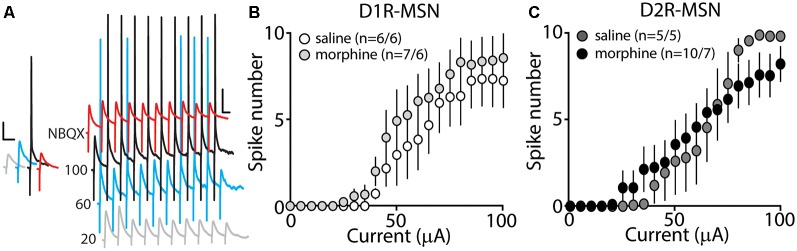
Synaptically-driven action potential firing on D1R- or D2R-MSNs is unaffected by repeated morphine (10 mg/kg, i.p.) treatment. **(A)** Representative traces showing depolarizations or action potentials of a recorded MSN evoked by electrical current (in μA) of 20 (light gray), 60 (blue), 100 (black), or 100 in the presence of NBQX (red), an AMPA receptor antagonist. Scale bars, 12.5 mV, 50 ms. **(B,C)** Summary graphs showing the average spike number at each current injected for D1R- or D2R-MSNs following saline or morphine treatment (cells/animals).

**Figure 6:**
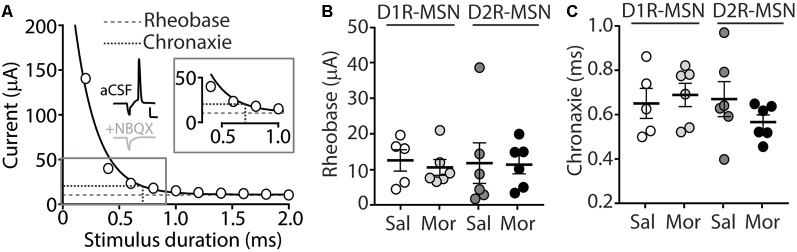
**(A)** A strength-duration curve constructed from an MSN in the nucleus accumbens shell. Stimulus current was adjusted at each duration (from 0 to 2.0 ms with 0.2 ms increments) until an action potential was evoked using an electrical stimulus. The curve was fit with a two-phase exponential decay. The rheobase (gray dashed line) was calculated as the plateau of the curve and the chronaxie (black dashed line) was calculated as 2× the rheobase (Inset). Representative traces illustrating the stimulus (downward deflection) followed by the action potential at 2 ms duration). In the presence of NBQX (2 μM), action potentials are not elicited (2 ms duration, 35 μA of current). Scale bars, 20 mV, 12.5 ms.** (B)** Summary graph showing the average rheobase for D1R- or D2R-MSNs following saline (Sal) or morphine (Mor; 10 mg/kg, i.p.) treatment. **(C)** Summary graph showing the average chronaxie for D1R- or D2R-MSNs following saline or morphine treatment.

Our present findings demonstrate that morphine exposure decreases mEPSC or mIPSC frequency and increases the intrinsic membrane excitability on D2R-MSNs. In an attempt to determine the integrated effect of these alterations on the overall functional output of D2R-MSNs, we measured the input-output efficacy by measuring synaptically-driven action potential firing (Hopf et al., 2003; Otaka et al., 2013). This was performed by counting the number of action potentials generated on D1R- or D2R-MSNs when varying currents (0–100 μA, 5 μA increments) were injected through a stimulating electrode during a 10 Hz stimulus. These measurements were performed in the absence of pharmacological blockers in the bath solution, thus cell-type-specific MSN responses were influenced by mixed excitatory and inhibitory inputs (see “Materials and Methods” section). Following morphine treatment, stimulating afferents in the nucleus accumbens elicited similar NBQX-sensitive action potential responses (Figure 5A) in D1R- (Figure 5B) or D2R-MSNs (Figure 5C) compared to saline controls (D1R-MSN: *F*_(20,220)_ = 0.349, *p* = 0.996; D2R-MSN: *F*_(20,260)_ = 1.05, *p* = 0.409, two-way repeated-measures ANOVA). Since neuronal excitability is not only influenced by the current intensity, but also by the temporal aspects of the current pulse, we constructed strength-duration curves whereby the electrically-evoked current was plotted over the electrically-evoked current duration (Figure 6). By constructing this curve, we were able to observe increases or decreases in pre- and postsynaptic connections shown as steep or shallow decays in amplitude, respectively, as the pulse duration increases (Fröhlich, 2016). Once plotted, the rheobase, minimal electrically stimulated current required to elicit an action potential at an infinite pulse duration, and the chronaxie, an indication of neuronal excitability defined by the duration of the stimulus corresponding to twice the rheobase, were calculated. Twenty-four hours following morphine treatment, we found that the rheobase was not significantly different compared to control conditions (*F*_(3,19)_ = 0.048, *p* = 0.986, one-way ANOVA; Figure 6B). Similarly, the chronaxie on D1R- or D2R-MSNs showed no significant change following morphine treatment (*F*_(3,19)_ = 0.8445, *p* = 0.486, one-way ANOVA; Figure 6C). Overall, these results suggest that the morphine-induced decreases in synaptic transmission on D2R-MSNs are countered by increases in intrinsic membrane excitability, which together, enable D2R-MSNs to maintain basal levels of functional output in response to synaptic input.

## Discussion

Our results show that repeated morphine administration preferentially alters action-potential independent synaptic transmission and intrinsic membrane excitability on D2R-MSNs, without affecting D1R-MSNs. Furthermore, our results show that the synaptically-driven action potential responses on D2R-MSNs, which are expected to integrate both synaptic and intrinsic cellular properties, remain unchanged following morphine exposure.

### Morphine-Induced Changes in MSN Intrinsic Membrane Excitability

A neuronal homeostatic response refers to a self-correcting property that is necessary in order to maintain stable function (Huang et al., 2011; Turrigiano, 2011). Here, our results show that 24 h post morphine treatment, the overall synaptic input on D2R-MSNs is reduced (Figures 1, 3), while the intrinsic membrane excitability is significantly increased (Figure 4). Given that the mammalian central nervous system, including the nucleus accumbens, is capable of compensatory changes in intrinsic membrane excitability to overcome attenuated synaptic function (Burrone et al., 2002; Maffei and Turrigiano, 2008; Ishikawa et al., 2009), it is possible that a homeostatic synaptic-to-membrane crosstalk enables D2R-MSNs to maintain sensitivity to incoming signals, which is supported by our observed non-significant change in functional output following morphine exposure (Figures 5, 6).

This potential homeostatic response to morphine is in line with observations in the nucleus accumbens, during short-term abstinence from repeated cocaine administration, whereby NMDA receptor synaptic expression is increased (Huang et al., 2009), while in parallel, MSN intrinsic membrane excitability is decreased (Zhang et al., 1998; Dong et al., 2006; Ishikawa et al., 2009; Kourrich and Thomas, 2009; Mu et al., 2010; Wang et al., 2018). Although, both cocaine and morphine elicit homeostatic compensatory changes, the contrasting effects on MSN synaptic transmission and intrinsic membrane excitability are potentially due to the drug’s cell-type-specific effects in the accumbens (Huang et al., 2009; Brown et al., 2011; Graziane et al., 2016).

Alternatively, homeostasis may not drive the opposing synaptic and intrinsic morphine-induced changes on D2R-MSNs as these changes may be two independent adaptations. Given that we observed morphine-induced reductions in both excitatory and inhibitory synaptic transmission on D2R-MSNs, the overall synaptic transmission may produce no net changes. This is supported by the sEPSC-to-sIPSC or the sIPCS-to-sEPSC inter-event intervals that show no change following morphine treatment (Figures 2D,E). These results suggest that the increases in the intrinsic membrane excitability would result in an overall excitation gain on D2R-MSNs. Although an excitation gain was not observed in our attempt to integrate both the morphine-induced synaptic and intrinsic properties (Figures 5, 6), it is possible that *in vivo* a more complicated scenario exists. Extensive evidence demonstrates that drugs of abuse influence the firing properties of neurons in the accumbens (Peoples et al., 1999; Carelli and Ijames, 2000; Ghitza et al., 2006; Calipari et al., 2016). Given that the bistable membrane potentials of MSNs (e.g., ~−80 mV downstate vs. ~−60 mV upstate; O’Donnell and Grace, 1995) are regulated by synaptic input and intrinsic factors (Plenz and Kitai, 1998; Wickens and Wilson, 1998; Huang et al., 2011), dysregulations in these factors may influence information flow from MSNs to downstream targets (O’Donnell et al., 1999), potentially influencing motivated behaviors.

Last, a previous study has shown that morphine exposure decreases the intrinsic membrane excitability on MSNs in the nucleus accumbens (NAc) with concomitant increases in the action potential amplitude, decreases in the action potential half-width, and decreases in the membrane resistance and tau (Heng et al., 2008). In contrast, following morphine exposure, we observed an increase in the intrinsic membrane excitability on D2R-MSNs in the NAc shell with no changes in action potential amplitude or half-width, increases in membrane resistance, and no changes in tau. These discrepancies may be a result of a number of differences between studies including the species (rat vs. mice), the recording location (unspecific recordings in the NAc vs. NAc shell), the exposure and recording paradigm (7 days morphine with recordings 3–4 days post-treatment vs. 5 days morphine with recordings 24 h post-treatment), and/or the bath temperature during recordings (30–32°C vs. 22–24°C). Regardless of this discrepancy, a key finding from our studies was the robust morphine-induced increase in intrinsic membrane excitability on D2R-MSNs, while the intrinsic membrane excitability on D1R-MSNs remained unaltered. These results demonstrate that morphine exposure produces cell-type-specific alterations within the reward neurocircuit.

### Excitatory-Inhibitory Balance

The spatiotemporal interaction between excitatory and inhibitory synaptic connections on a targeted neuron regulates neuronal activity (Wehr and Zador, 2003; Zerlaut and Destexhe, 2017; He and Cline, 2019), modulates neuronal oscillations (Buzsáki and Wang, 2012), and balances network dynamics (van Vreeswijk and Sompolinsky, 1996; Berke, 2009; Buzsáki and Watson, 2012; Denève and Machens, 2016; Bonnefond et al., 2017). A problem arises when the E-I balance is disrupted causing a chronic deviation from the original set-point, which is associated with pathological states including autism, schizophrenia, epilepsy, and addiction-like behaviors (Rubenstein and Merzenich, 2003; Eichler and Meier, 2008; Fritschy, 2008; Yizhar et al., 2011; Tejeda et al., 2017; Yu et al., 2017). Here, we investigated whether morphine abstinence alters the E/I ratio on D1R- or D2R-MSNs in the accumbens shell by comparing the evoked excitatory to inhibitory current amplitudes as well as the temporal integration of spontaneous excitatory and inhibitory events. We show that despite the morphine-induced changes in mEPSC and mIPSC frequency on D2R-MSNs, the E/I evoked current amplitude ratio and the temporal relationship between spontaneous excitatory and inhibitory events were unchanged following morphine administration (Figure 2), potentially due to homeostatic mechanisms that tightly maintain neuronal E-I balance on D2R-MSNs (Turrigiano and Nelson, 2000, 2004; Turrigiano, 2011).

We have not examined the mechanisms triggering the potential homeostatic mechanisms that maintain the E-I balance. However, previously, it has been shown that morphine-induced decreases in glutamatergic transmission on D2R-MSNs are prevented by administration of the GluA_2_3Y peptide, which prevents morphine-induced AMPAR removal from excitatory synapses (Ahmadian et al., 2004; Brebner et al., 2005; Wang, 2008; Graziane et al., 2016; Madayag et al., 2019). Future studies can investigate the synaptic cascade that potentially leads to the maintenance of the E-I balance on D2R-MSNs following morphine exposure, by administering GluA_2_3Y peptide and measuring effects on D2R-MSN mIPSC frequency and intrinsic membrane excitability. Such work may reveal a homeostatic mechanism triggered by morphine-induced decreases in glutamatergic synaptic transmission that may also regulate intrinsic membrane excitability.

Last, we observed a non-significant change in functional output on D2R-MSNs following morphine exposure (Figures 5, 6) despite the increases in intrinsic membrane excitability (Figure 4). This result is potentially explained by our synaptic assessments showing an overall decrease in mEPSC frequency, mIPSC frequency, with no change on the mEPSC or mIPSC amplitude, E/I ratio, or on the temporal relationship between the E-I balance (Figure 2). These results suggest that, following morphine exposure, the overall somatic summation of excitatory and inhibitory currents on D2R-MSNs is potentially weakened. This is likely caused by weakened postsynaptic excitatory glutamatergic synaptic connections [i.e., decreases in mEPSC frequency (Figure 1) and increases in the expression of silent synapses (Graziane et al., 2016)] as well as the alterations in presynaptic factors that result in decreased inhibitory synaptic transmission [i.e., decreases in mIPSC frequency (Figure 3)]. These morphine-induced decreases in synaptic transmission on D2R-MSNs along with the morphine-induced increases in intrinsic membrane excitability, together, likely enable D2R-MSNs to maintain basal levels of functional output in response to synaptic input.

### Receptor Kinetics

AMPA/kainate receptor kinetics comprise a rapidly rising conductance that decays as the agonist-receptor complex deactivates (Traynelis et al., 2010). This process is regulated by a number of factors including receptor subunit composition (Sommer et al., 1990; Partin et al., 1996; Quirk et al., 2004) and auxiliary regulatory proteins (Milstein et al., 2007; Milstein and Nicoll, 2008). Here, we show that 24 h post morphine treatment, the AMPA/kainate receptor kinetics (rise time and decay tau) on D1R- or D2R-MSNs are unchanged (Figures 1H,I). This result cannot exclude potential alterations in morphine-induced auxiliary protein expression or receptor subunit composition. It has been shown that mRNAs for AMPA receptor subunits GluA1, 3, and 4 are significantly decreased in morphine self-administering rats (Hemby, 2004). However, on average, any morphine-induced changes that may occur, are unable to elicit changes in overall kinetic properties of AMPA/kainate receptors responding to action potential independent glutamate release. This suggests that if morphine-induces any potential changes in EPSC temporal summation at the soma, these alterations are likely not mediated by changes in AMPA/kainate receptor kinetics. Similarly, we observed no changes in inhibitory ionotropic neurotransmitter receptor kinetics on D1R- or D2R-MSNs following morphine treatment (Figures 3H,I), again suggesting that, overall, if morphine was able to induce changes in receptor phosphorylation, density, or scaffolding proteins, factors regulating inhibitory ionotropic receptor kinetics (Verdoorn et al., 1990; Takahashi et al., 1992; Tia et al., 1996; Jones and Westbrook, 1997; Chen et al., 2000), they are unable to influence the overall kinetic properties of inhibitory ionotropic receptors responding to action potential independent neurotransmitter release.

### Sex Comparisons

We found that, within all measurements where male and female mice were used (e.g., mEPSC recordings, mIPSC recordings, E/I ratios, intrinsic membrane excitability, synaptically-driven action potentials, and rheobase/chronaxie measurements), there were no statistically significant sex differences within D1R- or D2R-MSNs following non-contingent, repeated saline or morphine treatment (Table 3). Because of this, animals were pooled. However, we understand that our statistical assessment is likely underpowered and therefore, future experiments are required to directly test sex differences. Additionally, it is possible that bimodal distributions in our data set are influenced by sex effects. For example, Figure 1G shows a bimodal distribution in the D1R-MSN cell population following morphine treatment. However, upon further analysis, these two populations consist of neurons from both males and females suggesting that at the 24 h abstinence time point following repeated morphine administration, mEPSC frequency on D1R-MSNs is unaltered. Despite this, it is still worthwhile to perform a thorough assessment of potential sex effects as it has been shown that, under basal conditions, D2R-MSN mEPSC frequency is significantly reduced in female vs. male prepubertal (2–3 weeks old) mice, in the accumbens core (Cao et al., 2018). Determining whether these sex differences are observed into adulthood following morphine exposure would be an interesting future direction.

**Table 3:** Sex comparisons within electrophysiological assessments investigating morphine-induced changes in synaptic and intrinsic properties of D1R- or D2R-MSNs.

Experiment	Saline D1R		*P*-value	Saline D2R		*P*-value	Morphine D1R		*P*-value	Morphine D2R		*P*-value
	Male	Female		Male	Female		Male	Female		Male	Female	
mEPSC Frequency (Figure 1)	6.67 ± 0.51 (6)	6.82 ± 0.68 (6)	0.86	6.32 ± 1.43 (5)	5.55 ± 1.40 (7)	0.71	5.17 ± 0.55 (4)	4.61 ± 0.61 (7)	0.58	3.33 ± 0.44 (8)	2.30 ± 0.28 (3)	0.21
mEPSC Amplitude (Figure 1)	15.12 ± 1.34 (6)	14.34 ± 1.14 (6)	0.67	12.48 ± 1.10 (5)	13.55 ± 1.07 (7)	0.51	14.58 ± 1.29 (4)	14.15 ± 0.83 (7)	0.78	12.28 ± 0.56 (8)	11.50 ± 0.51 (3)	0.46
E/I Ratio(Figure 2)	0.22 ± 0.06 (2)	0.37 ± 0.10 (9)	0.50	0.28 ± 0.05 (2)	0.46 ± 0.14 (7)	0.52	0.25 ± 0.10 (3)	0.38 ± 0.09 (8)	0.47	0.09 ± 0.03 (2)	0.32 ± 0.06 (8)	0.12
mIPSC Frequency (Figure 3)	1.10 ± 0.17 (5)	1.41 ± 0.18 (13)	0.35	1.55 ± 0.21 (4)	1.97 ± 0.20 (10)	0.26	1.21 ± 0.15 (5)	1.03 ± 0.12 (12)	0.43	0.91 ± 0.12 (4)	1.05 ± 0.10 (16)	0.50
mIPSC Amplitude (Figure 3)	31.44 ± 2.46 (5)	31.39 ± 1.94 (13)	0.99	34.20 ± 3.91 (4)	35.57 ± 4.30 (10)	0.85	30.62 ± 2.09 (5)	28.09 ± 2.63 (12)	0.57	33.27 ± 4.57 (4)	41.07 ± 2.47 (16)	0.17
IME (450pA) (Figure 4)	2.43 ± 0.20 (7)	2.75 ± 0.48 (4)	0.49	4.29 ± 0.97 (7)	2.50 ± 0.29 (4)	0.21	2.86 ± 0.40 (7)	4.20 ± 1.36 (5)	0.30	5.67 ± 0.88 (6)	6.38 ± 0.65 (8)	0.52

*Number of cells (n). Student’s *t*-test was used for statistical measures. Abbreviations: mEPS, Cminiature excitatory postsynatpic current; E/I ratio, excitatory to inhibitory ratio; mIPSC, miniature inhibitory postsynaptic current; IME, intrinsic membrane excitability*.

### Cell Type Comparisons

The nucleus accumbens is a complicated network consisting of D1R and D2R-MSNs that project to similar brain regions (Smith et al., 2013). Additionally, it has been shown that lateral inhibition between MSNs exists and this lateral inhibition is critically involved in addiction-like behaviors (Dobbs et al., 2016). Therefore, imbalances in D1R- and D2R-MSN activity, in downstream targets, or within the accumbens microcircuit, are potentially responsible for behavioral phenotypes (e.g., locomotor activity or conditioned place preference) observed following repeated morphine treatment (Zarrindast et al., 2002; Bohn et al., 2003; Tzschentke, 2007). Using our statistical approach, we found a significant difference in mIPSC amplitude between D1R-MSN morphine and D2R-MSNs morphine (*F*_(3,65)_ = 4.73, *p* = 0.005; one way ANOVA with Bonferroni post-test revealing significant differences between D1R-MSN morphine and D2R-MSN morphine, *p* = 0.0048; Figure 3D). This result provides a potentially interesting opportunity to determine whether significant differences in electrophysiological readouts between neuronal types is sufficient to contribute to drug-induced behavioral phenotypes. For example, increasing D1R-MSN mIPSC amplitude or decreasing D2R-MSN mIPSC amplitude in morphine-treated animals may block morphine-induced behavioral phenotypes as cell-type interactions may drive morphine-induced behaviors. This idea may also be applied to our observed significant difference in mIPSC frequency between D1R- and D2R-MSNs in saline-treated animals (*p* = 0.024, Bonferroni post-test), which was non-significant following morphine treatment (Figure 3G). Based on these cell-type-specific comparisons in electrophysiological data, it will be interesting to test whether cell-type-specific interactions significantly contribute to addiction-like behaviors.

## Conclusion

In conclusion, this study demonstrates new information on how morphine exposure alters both extrinsic and intrinsic neuronal properties of MSNs in the nucleus accumbens shell. The alterations observed on D2R-MSNs appear to be opposing in nature, resulting in a maintenance of basal levels of functional output. It is known that preventing morphine-induced decreases in glutamatergic transmission on D2R-MSNs blocks the prolonged maintenance (21 days post-conditioning) of morphine-induced CPP (Graziane et al., 2016). Therefore, it is plausible that morphine-induced alterations on synaptic and intrinsic excitability of D2R-MSNs may not alter D2R-MSN output during short-term abstinence, but may instead result in an allostatic set point of excitability that results in long-term behavioral consequences (Koob and Le Moal, 2001). Although, future studies are required to directly test whether the observed maintenance of D2R-MSN output drives the prolonged-expression of opioid-seeking behaviors.

## Data Availability Statement

The datasets generated for this study are available on request to the corresponding author.

## Ethics Statement

The animal study was reviewed and approved by Pennsylvania State University College of Medicine Institutional Animal Care and Use Committee.

## Author Contributions

DM and NG designed the experiments and analyses, conducted the experiments and data analyses, and wrote the manuscript. BJ designed a program for the analysis performed in Figure 2.

## Conflict of Interest

The authors declare that the research was conducted in the absence of any commercial or financial relationships that could be construed as a potential conflict of interest.

## References

[B1] Ade K. K. Wan Y. Chen M. Gloss B. Calakos N. (2011). An improved BAC transgenic fluorescent reporter line for sensitive and specific identification of striatonigral medium spiny neurons. Front. Syst. Neurosci. 5:32. 10.3389/fnsys.2011.00032 21713123 PMC3113108

[B2] Ahmadian G. Ju W. Liu L. Wyszynski M. Lee S. H. Dunah A. W.. (2004). Tyrosine phosphorylation of GluR2 is required for insulin-stimulated AMPA receptor endocytosis and LTD. EMBO J. 23, 1040–1050. 10.1038/sj.emboj.7600126 14976558 PMC380981

[B3] Al-Hasani R. McCall J. G. Shin G. Gomez A. M. Schmitz G. P. Bernardi J. M.. (2015). Distinct subpopulations of nucleus accumbens dynorphin neurons drive aversion and reward. Neuron 87, 1063–1077. 10.1016/j.neuron.2015.08.019 26335648 PMC4625385

[B4] Berke J. D. (2009). Fast oscillations in cortical-striatal networks switch frequency following rewarding events and stimulant drugs. Eur. J. Neurosci. 30, 848–859. 10.1111/j.1460-9568.2009.06843.x 19659455 PMC2778242

[B5] Bertran-Gonzalez J. Bosch C. Maroteaux M. Matamales M. Hervé D. Valjent E.. (2008). Opposing patterns of signaling activation in dopamine D_1_ and D_2_ receptor-expressing striatal neurons in response to cocaine and haloperidol. J. Neurosci. 28, 5671–5685. 10.1523/JNEUROSCI.1039-08.2008 18509028 PMC6670792

[B6] Bhatia A. Moza S. Bhalla U. S. (2019). Precise excitation-inhibition balance controls gain and timing in the hippocampus. Elife 8:e43415. 10.7554/eLife.43415 31021319 PMC6517031

[B7] Bohn L. M. Gainetdinov R. R. Sotnikova T. D. Medvedev I. O. Lefkowitz R. J. Dykstra L. A.. (2003). Enhanced rewarding properties of morphine, but not cocaine, in β(arrestin)-2 knock-out mice. J. Neurosci. 23, 10265–10273. 10.1523/JNEUROSCI.23-32-10265.2003 14614085 PMC6741024

[B8] Bonnefond M. Kastner S. Jensen O. (2017). Communication between brain areas based on nested oscillations. eNeuro 4:ENEURO.0153-16.2017. 10.1523/eneuro.0153-16.2017 28374013 PMC5367085

[B9] Brebner K. Wong T. P. Liu L. Liu Y. Campsall P. Gray S.. (2005). Nucleus accumbens long-term depression and the expression of behavioral sensitization. Science 310, 1340–1343. 10.1126/science.1116894 16311338

[B10] Brown T. E. Lee B. R. Mu P. Ferguson D. Dietz D. Ohnishi Y. N.. (2011). A silent synapse-based mechanism for cocaine-induced locomotor sensitization. J. Neurosci. 31, 8163–8174. 10.1523/JNEUROSCI.0016-11.2011 21632938 PMC3286116

[B11] Burrone J. O’Byrne M. Murthy V. N. (2002). Multiple forms of synaptic plasticity triggered by selective suppression of activity in individual neurons. Nature 420, 414–418. 10.1038/nature01242 12459783

[B12] Buzsáki G. Wang X. J. (2012). Mechanisms of γ oscillations. Annu. Rev. Neurosci. 35, 203–225. 10.1146/annurev-neuro-062111-150444 22443509 PMC4049541

[B13] Buzsáki G. Watson B. O. (2012). Brain rhythms and neural syntax: implications for efficient coding of cognitive content and neuropsychiatric disease. Dialogues Clin. Neurosci. 14, 345–367. 23393413 10.31887/DCNS.2012.14.4/gbuzsaki PMC3553572

[B14] Calipari E. S. Bagot R. C. Purushothaman I. Davidson T. J. Yorgason J. T. Pena C. J.. (2016). *In vivo* imaging identifies temporal signature of D1 and D2 medium spiny neurons in cocaine reward. Proc. Natl. Acad. Sci. U S A 113, 2726–2731. 10.1073/pnas.1521238113 26831103 PMC4791010

[B15] Cao J. Dorris D. M. Meitzen J. (2018). Electrophysiological properties of medium spiny neurons in the nucleus accumbens core of prepubertal male and female Drd1a-tdTomato line 6 BAC transgenic mice. J. Neurophysiol. 120, 1712–1727. 10.1152/jn.00257.2018 29975170 PMC6230806

[B16] Carelli R. M. Ijames S. G. (2000). Nucleus accumbens cell firing during maintenance, extinction, and reinstatement of cocaine self-administration behavior in rats. Brain Res. 866, 44–54. 10.1016/s0006-8993(00)02217-4 10825479

[B17] Castro D. C. Bruchas M. R. (2019). A motivational and neuropeptidergic hub: anatomical and functional diversity within the nucleus accumbens shell. Neuron 102, 529–552. 10.1016/j.neuron.2019.03.003 31071288 PMC6528838

[B18] Chen L. Wang H. Vicini S. Olsen R. W. (2000). The γ-aminobutyric acid type A (GABA_A_) receptor-associated protein (GABARAP) promotes GABA_A_ receptor clustering and modulates the channel kinetics. Proc. Natl. Acad. Sci. U S A 97, 11557–11562. 10.1073/pnas.190133497 10984509 PMC17239

[B19] Creed M. Ntamati N. R. Chandra R. Lobo M. K. Luscher C. (2016). Convergence of reinforcing and anhedonic cocaine effects in the ventral pallidum. Neuron 92, 214–226. 10.1016/j.neuron.2016.09.001 27667004 PMC8480039

[B20] Denève S. Machens C. K. (2016). Efficient codes and balanced networks. Nat. Neurosci. 19, 375–382. 10.1038/nn.4243 26906504

[B21] Desai N. S. Rutherford L. C. Turrigiano G. G. (1999). Plasticity in the intrinsic excitability of cortical pyramidal neurons. Nat. Neurosci. 2, 515–520. 10.1038/9165 10448215

[B22] Dobbs L. K. Kaplan A. R. Lemos J. C. Matsui A. Rubinstein M. Alvarez V. A. (2016). Dopamine regulation of lateral inhibition between striatal neurons gates the stimulant actions of cocaine. Neuron 90, 1100–1113. 10.1016/j.neuron.2016.04.031 27181061 PMC4891261

[B23] Dong Y. Green T. Saal D. Marie H. Neve R. Nestler E. J.. (2006). CREB modulates excitability of nucleus accumbens neurons. Nat. Neurosci. 9, 475–477. 10.1038/nn1661 16520736

[B24] Eichler S. A. Meier J. C. (2008). E-I balance and human diseases—from molecules to networking. Front. Mol. Neurosci. 1:2. 10.3389/neuro.02.002.2008 18946535 PMC2526001

[B25] Enoksson T. Bertran-Gonzalez J. Christie M. J. (2012). Nucleus accumbens D2- and D1-receptor expressing medium spiny neurons are selectively activated by morphine withdrawal and acute morphine, respectively. Neuropharmacology 62, 2463–2471. 10.1016/j.neuropharm.2012.02.020 22410393

[B26] Figl T. Lewis T. Barry P. (2003). Liquid junction potential corrections. AxoBits [Online] 39, 6–10. Available online at: https://medicalsciences.med.unsw.edu.au/sites/default/files/soms/page/ElectroPhysSW/LJP_article_%20AxoBits%2039.pdf

[B27] Fritschy J. M. (2008). Epilepsy, E/I balance and GABA_A_ receptor plasticity. Front. Mol. Neurosci. 1:5. 10.3389/neuro.02.005.2008 18946538 PMC2525999

[B28] Fröhlich F. (2016). “Chapter 14—deep brain stimulation,” in Network Neuroscience, ed. Fröhlich F. (San Diego, CA: Academic Press), 187–196.

[B29] Ghitza U. E. Prokopenko V. F. West M. O. Fabbricatore A. T. (2006). Higher magnitude accumbal phasic firing changes among core neurons exhibiting tonic firing increases during cocaine self-administration. Neuroscience 137, 1075–1085. 10.1016/j.neuroscience.2005.10.026 16325346

[B30] Graziane N. M. Sun S. Wright W. J. Jang D. Liu Z. Huang Y. H.. (2016). Opposing mechanisms mediate morphine- and cocaine-induced generation of silent synapses. Nat. Neurosci. 19, 915–925. 10.1038/nn.4313 27239940 PMC4925174

[B31] Hanse E. Seth H. Riebe I. (2013). AMPA-silent synapses in brain development and pathology. Nat. Rev. Neurosci. 14, 839–850. 10.1038/nrn3642 24201185

[B32] He H. Y. Cline H. T. (2019). What is excitation/inhibition and how is it regulated? A case of the elephant and the wisemen. J. Exp. Neurosci. 13:1179069519859371. 10.1177/1179069519859371 31258334 PMC6591655

[B33] He H. Y. Shen W. Zheng L. Guo X. Cline H. T. (2018). Excitatory synaptic dysfunction cell-autonomously decreases inhibitory inputs and disrupts structural and functional plasticity. Nat. Commun. 9:2893. 10.1038/s41467-018-05125-4 30042473 PMC6057951

[B34] Hearing M. Graziane N. Dong Y. Thomas M. J. (2018). Opioid and psychostimulant plasticity: targeting overlap in nucleus accumbens glutamate signaling. Trends Pharmacol. Sci. 39, 276–294. 10.1016/j.tips.2017.12.004 29338873 PMC5818297

[B35] Hearing M. C. Jedynak J. Ebner S. R. Ingebretson A. Asp A. J. Fischer R. A.. (2016). Reversal of morphine-induced cell-type-specific synaptic plasticity in the nucleus accumbens shell blocks reinstatement. Proc. Natl. Acad. Sci. U S A 113, 757–762. 10.1073/pnas.1519248113 26739562 PMC4725472

[B36] Heinsbroek J. A. Neuhofer D. N. Griffin W. C. III. Siegel G. S. Bobadilla A.-C. Kupchik Y. M.. (2017). Loss of plasticity in the D2-accumbens pallidal pathway promotes cocaine seeking. J. Neurosci. 37, 757–767. 10.1523/JNEUROSCI.2659-16.2016 28123013 PMC5296778

[B37] Hemby S. E. (2004). Morphine-induced alterations in gene expression of calbindin immunopositive neurons in nucleus accumbens shell and core. Neuroscience 126, 689–703. 10.1016/j.neuroscience.2004.01.056 15183518

[B38] Heng L. J. Yang J. Liu Y. H. Wang W. T. Hu S. J. Gao G. D. (2008). Repeated morphine exposure decreased the nucleus accumbens excitability during short-term withdrawal. Synapse 62, 775–782. 10.1002/syn.20551 18655119

[B39] Higley M. J. Contreras D. (2006). Balanced excitation and inhibition determine spike timing during frequency adaptation. J. Neurosci. 26, 448–457. 10.1523/JNEUROSCI.3506-05.2006 16407542 PMC6674406

[B40] Hikida T. Kimura K. Wada N. Funabiki K. Nakanishi S. (2010). Distinct roles of synaptic transmission in direct and indirect striatal pathways to reward and aversive behavior. Neuron 66, 896–907. 10.1016/j.neuron.2010.05.011 20620875

[B41] Hiratani N. Fukai T. (2017). Detailed dendritic excitatory/inhibitory balance through heterosynaptic spike-timing-dependent plasticity. J. Neurosci. 37, 12106–12122. 10.1523/JNEUROSCI.0027-17.2017 29089443 PMC6596817

[B42] Hopf F. W. Cascini M. G. Gordon A. S. Diamond I. Bonci A. (2003). Cooperative activation of dopamine D1 and D2 receptors increases spike firing of nucleus accumbens neurons *via* G-protein βγ subunits. J. Neurosci. 23, 5079–5087. 10.1523/JNEUROSCI.23-12-05079.2003 12832531 PMC6741176

[B43] Huang Y. H. Lin Y. Mu P. Lee B. R. Brown T. E. Wayman G.. (2009). *in vivo* cocaine experience generates silent synapses. Neuron 63, 40–47. 10.1016/j.neuron.2009.06.007 19607791 PMC2721479

[B44] Huang Y. H. Schluter O. M. Dong Y. (2011). Cocaine-induced homeostatic regulation and dysregulation of nucleus accumbens neurons. Behav. Brain Res. 216, 9–18. 10.1016/j.bbr.2010.07.039 20708038 PMC2975799

[B45] Ishikawa M. Mu P. Moyer J. T. Wolf J. A. Quock R. M. Davies N. M.. (2009). Homeostatic synapse-driven membrane plasticity in nucleus accumbens neurons. J. Neurosci. 29, 5820–5831. 10.1523/JNEUROSCI.5703-08.2009 19420249 PMC2743333

[B46] Jones M. V. Westbrook G. L. (1997). Shaping of IPSCs by endogenous calcineurin activity. J. Neurosci. 17, 7626–7633. 10.1523/JNEUROSCI.17-20-07626.1997 9315884 PMC6793922

[B47] Kerchner G. A. Nicoll R. A. (2008). Silent synapses and the emergence of a postsynaptic mechanism for LTP. Nat. Rev. Neurosci. 9, 813–825. 10.1038/nrn2501 18854855 PMC2819160

[B48] Koo J. W. Lobo M. K. Chaudhury D. Labonté B. Friedman A. Heller E.. (2014). Loss of BDNF signaling in D1R-expressing NAc neurons enhances morphine reward by reducing GABA inhibition. Neuropsychopharmacology 39, 2646–2653. 10.1038/npp.2014.118 24853771 PMC4207344

[B49] Koob G. F. Le Moal M. (2001). Drug addiction, dysregulation of reward, and allostasis. Neuropsychopharmacology 24, 97–129. 10.1016/s0893-133x(00)00195-0 11120394

[B50] Kourrich S. Thomas M. J. (2009). Similar neurons, opposite adaptations: psychostimulant experience differentially alters firing properties in accumbens core versus shell. J. Neurosci. 29, 12275–12283. 10.1523/JNEUROSCI.3028-09.2009 19793986 PMC3307102

[B51] Kourrich S. Calu D. J. Bonci A. (2015). Intrinsic plasticity: an emerging player in addiction. Nat. Rev. Neurosci. 16, 173–184. 10.1038/nrn3877 25697160

[B52] Lalchandani R. R. van der Goes M.-S. Partridge J. G. Vicini S. (2013). Dopamine D_2_ receptors regulate collateral inhibition between striatal medium spiny neurons. J. Neurosci. 33, 14075–14086. 10.1523/JNEUROSCI.0692-13.2013 23986243 PMC3756755

[B53] Lee B. R. Ma Y. Y. Huang Y. H. Wang X. Otaka M. Ishikawa M.. (2013). Maturation of silent synapses in amygdala-accumbens projection contributes to incubation of cocaine craving. Nat. Neurosci. 16, 1644–1651. 10.1038/nn.3533 24077564 PMC3815713

[B54] Liu Z. Wang Y. Cai L. Li Y. Chen B. Dong Y.. (2016). Prefrontal cortex to accumbens projections in sleep regulation of reward. J. Neurosci. 36, 7897–7910. 10.1523/JNEUROSCI.0347-16.2016 27466335 PMC4961777

[B55] Lobo M. K. Covington H. E. III. Chaudhury D. Friedman A. K. Sun H. Damez-Werno D.. (2010). Cell type-specific loss of BDNF signaling mimics optogenetic control of cocaine reward. Science 330, 385–390. 10.1126/science.1188472 20947769 PMC3011229

[B56] Madayag A. C. Gomez D. Anderson E. M. Ingebretson A. E. Thomas M. J. Hearing M. C. (2019). Cell-type and region-specific nucleus accumbens AMPAR plasticity associated with morphine reward, reinstatement, and spontaneous withdrawal. Brain Struct. Funct. 224, 2311–2324. 10.1007/s00429-019-01903-y 31201496 PMC6698404

[B57] Maffei A. Turrigiano G. G. (2008). Multiple modes of network homeostasis in visual cortical layer 2/3. J. Neurosci. 28, 4377–4384. 10.1523/JNEUROSCI.5298-07.2008 18434516 PMC2655203

[B58] McDevitt D. S. Graziane N. M. (2018). Neuronal mechanisms mediating pathological reward-related behaviors: a focus on silent synapses in the nucleus accumbens. Pharmacol. Res. 136, 90–96. 10.1016/j.phrs.2018.08.025 30171902

[B59] Milstein A. D. Nicoll R. A. (2008). Regulation of AMPA receptor gating and pharmacology by TARP auxiliary subunits. Trends Pharmacol. Sci. 29, 333–339. 10.1016/j.tips.2008.04.004 18514334 PMC2819157

[B60] Milstein A. D. Zhou W. Karimzadegan S. Bredt D. S. Nicoll R. A. (2007). TARP subtypes differentially and dose-dependently control synaptic AMPA receptor gating. Neuron 55, 905–918. 10.1016/j.neuron.2007.08.022 17880894 PMC3167227

[B61] Mu P. Moyer J. T. Ishikawa M. Zhang Y. Panksepp J. Sorg B. A.. (2010). Exposure to cocaine dynamically regulates the intrinsic membrane excitability of nucleus accumbens neurons. J. Neurosci. 30, 3689–3699. 10.1523/JNEUROSCI.4063-09.2010 20220002 PMC2853189

[B62] Nelson A. B. Krispel C. M. Sekirnjak C. Du Lac S. (2003). Long-lasting increases in intrinsic excitability triggered by inhibition. Neuron 40, 609–620. 10.1016/s0896-6273(03)00641-x 14642283

[B63] O’Donnell P. Grace A. A. (1995). Synaptic interactions among excitatory afferents to nucleus accumbens neurons: hippocampal gating of prefrontal cortical input. J. Neurosci. 15, 3622–3639. 10.1523/jneurosci.15-05-03622.1995 7751934 PMC6578219

[B64] O’Donnell P. Greene J. Pabello N. Lewis B. L. Grace A. A. (1999). Modulation of cell firing in the nucleus accumbens. Ann. N Y Acad. Sci. 877, 157–175. 10.1111/j.1749-6632.1999.tb09267.x 10415649

[B65] Okun M. Lampl I. (2008). Instantaneous correlation of excitation and inhibition during ongoing and sensory-evoked activities. Nat. Neurosci. 11:535. 10.1038/nn.2105 18376400

[B66] Otaka M. Ishikawa M. Lee B. R. Liu L. Neumann P. A. Cui R.. (2013). Exposure to cocaine regulates inhibitory synaptic transmission in the nucleus accumbens. J. Neurosci. 33, 6753–6758. 10.1523/JNEUROSCI.4577-12.2013 23595733 PMC3661009

[B67] Partin K. M. Fleck M. W. Mayer M. L. (1996). AMPA receptor flip/flop mutants affecting deactivation, desensitization and modulation by cyclothiazide, aniracetam and thiocyanate. J. Neurosci. 16, 6634–6647. 10.1523/jneurosci.16-21-06634.1996 8824304 PMC6579261

[B68] Paxinos G. Franklin K. B. J. (2004). The Mouse Brain in Stereotaxic Coordinates. Elsevier: Academic Press.

[B69] Peoples L. L. Uzwiak A. J. Gee F. West M. O. (1999). Tonic firing of rat nucleus accumbens neurons: changes during the first 2 weeks of daily cocaine self-administration sessions. Brain Res. 822, 231–236. 10.1016/s0006-8993(98)01271-2 10082901

[B70] Pernía-Andrade A. J. Goswami S. P. Stickler Y. Frobe U. Schlogl A. Jonas P. (2012). A deconvolution-based method with high sensitivity and temporal resolution for detection of spontaneous synaptic currents *in vitro* and *in vivo*. Biophys. J. 103, 1429–1439. 10.1016/j.bpj.2012.08.039 23062335 PMC3471482

[B71] Plenz D. Kitai S. T. (1998). Up and down states in striatal medium spiny neurons simultaneously recorded with spontaneous activity in fast-spiking interneurons studied in cortex-striatum-substantia nigra organotypic cultures. J. Neurosci. 18, 266–283. 10.1523/JNEUROSCI.18-01-00266.1998 9412506 PMC6793428

[B72] Quirk J. C. Siuda E. R. Nisenbaum E. S. (2004). Molecular determinants responsible for differences in desensitization kinetics of AMPA receptor splice variants. J. Neurosci. 24, 11416–11420. 10.1523/JNEUROSCI.2464-04.2004 15601947 PMC6730378

[B73] Roland P. E. Bonde L. H. Forsberg L. E. Harvey M. A. (2017). Breaking the excitation-inhibition balance makes the cortical network’s space-time dynamics distinguish simple visual scenes. Front. Syst. Neurosci. 11:14. 10.3389/fnsys.2017.00014 28377701 PMC5360108

[B74] Rubenstein J. L. Merzenich M. M. (2003). Model of autism: increased ratio of excitation/inhibition in key neural systems. Genes Brain Behav. 2, 255–267. 10.1034/j.1601-183x.2003.00037.x 14606691 PMC6748642

[B75] Russo S. J. Dietz D. M. Dumitriu D. Morrison J. H. Malenka R. C. Nestler E. J. (2010). The addicted synapse: mechanisms of synaptic and structural plasticity in nucleus accumbens. Trends Neurosci. 33, 267–276. 10.1016/j.tins.2010.02.002 20207024 PMC2891948

[B76] Salgado S. Kaplitt M. G. (2015). The nucleus accumbens: a comprehensive review. Stereotact. Funct. Neurosurg. 93, 75–93. 10.1159/000368279 25720819

[B77] Scofield M. Heinsbroek J. Gipson C. Kupchik Y. Spencer S. Smith A.. (2016). The nucleus accumbens: mechanisms of addiction across drug classes reflect the importance of glutamate homeostasis. Pharmacol. Rev. 68, 816–871. 10.1124/pr.116.012484 27363441 PMC4931870

[B78] Sesack S. R. Grace A. A. (2010). Cortico-basal ganglia reward network: microcircuitry. Neuropsychopharmacology 35, 27–47. 10.1038/npp.2009.93 19675534 PMC2879005

[B79] Smith R. J. Lobo M. K. Spencer S. Kalivas P. W. (2013). Cocaine-induced adaptations in D1 and D2 accumbens projection neurons (a dichotomy not necessarily synonymous with direct and indirect pathways). Curr. Opin. Neurobiol. 23, 546–552. 10.1016/j.conb.2013.01.026 23428656 PMC3681928

[B80] Sommer B. Keinanen K. Verdoorn T. A. Wisden W. Burnashev N. Herb A.. (1990). Flip and flop: a cell-specific functional switch in glutamate-operated channels of the CNS. Science 249, 1580–1585. 10.1126/science.1699275 1699275

[B81] Takahashi T. Momiyama A. Hirai K. Hishinuma F. Akagi H. (1992). Functional correlation of fetal and adult forms of glycine receptors with developmental changes in inhibitory synaptic receptor channels. Neuron 9, 1155–1161. 10.1016/0896-6273(92)90073-m 1281418

[B82] Tejeda H. A. Wu J. Kornspun A. R. Pignatelli M. Kashtelyan V. Krashes M. J.. (2017). Pathway- and cell-specific κ-opioid receptor modulation of excitation-inhibition balance differentially gates D1 and D2 accumbens neuron activity. Neuron 93, 147–163. 10.1016/j.neuron.2016.12.005 28056342 PMC5808882

[B83] Thibault D. Loustalot F. Fortin G. M. Bourque M.-J. Trudeau L.-É. (2013). Evaluation of D1 and D2 dopamine receptor segregation in the developing striatum using BAC transgenic mice. PLoS One 8:e67219. 10.1371/journal.pone.0067219 23843993 PMC3699584

[B84] Tia S. Wang J. F. Kotchabhakdi N. Vicini S. (1996). Distinct deactivation and desensitization kinetics of recombinant GABA_A_ receptors. Neuropharmacology 35, 1375–1382. 10.1016/s0028-3908(96)00018-4 9014154

[B85] Traynelis S. F. Wollmuth L. P. Mcbain C. J. Menniti F. S. Vance K. M. Ogden K. K.. (2010). Glutamate receptor ion channels: structure, regulation and function. Pharmacol. Rev. 62, 405–496. 10.1124/pr.109.002451 20716669 PMC2964903

[B86] Turrigiano G. (2011). Too many cooks? Intrinsic and synaptic homeostatic mechanisms in cortical circuit refinement. Annu. Rev. Neurosci. 34, 89–103. 10.1146/annurev-neuro-060909-153238 21438687

[B87] Turrigiano G. G. Nelson S. B. (2000). Hebb and homeostasis in neuronal plasticity. Curr. Opin. Neurobiol. 10, 358–364. 10.1016/s0959-4388(00)00091-x 10851171

[B88] Turrigiano G. G. Nelson S. B. (2004). Homeostatic plasticity in the developing nervous system. Nat. Rev. Neurosci. 5, 97–107. 10.1038/nrn1327 14735113

[B89] Tzschentke T. M. (2007). Measuring reward with the conditioned place preference (CPP) paradigm: update of the last decade. Addict. Biol. 12, 227–462. 10.1111/j.1369-1600.2007.00070.x 17678505

[B90] van Vreeswijk C. Sompolinsky H. (1996). Chaos in neuronal networks with balanced excitatory and inhibitory activity. Science 274, 1724–1726. 10.1126/science.274.5293.1724 8939866

[B91] Verdoorn T. A. Draguhn A. Ymer S. Seeburg P. H. Sakmann B. (1990). Functional properties of recombinant rat GABA_A_ receptors depend upon subunit composition. Neuron 4, 919–928. 10.1016/0896-6273(90)90145-6 1694446

[B93] Wang Y. T. (2008). Probing the role of AMPAR endocytosis and long-term depression in behavioural sensitization: relevance to treatment of brain disorders, including drug addiction. Br. J. Pharmacol. 153, S389–395. 10.1038/sj.bjp.0707616 18059315 PMC2268058

[B92] Wang J. Ishikawa M. Yang Y. Otaka M. Kim J. Y. Gardner G. R.. (2018). Cascades of homeostatic dysregulation promote incubation of cocaine craving. J. Neurosci. 38, 4316–4328. 10.1523/JNEUROSCI.3291-17.2018 29626166 PMC5932642

[B94] Wehr M. Zador A. M. (2003). Balanced inhibition underlies tuning and sharpens spike timing in auditory cortex. Nature 426, 442–446. 10.1038/nature02116 14647382

[B95] Wickens J. R. Wilson C. J. (1998). Regulation of action-potential firing in spiny neurons of the rat neostriatum *in vivo*. J. Neurophysiol. 79, 2358–2364. 10.1152/jn.1998.79.5.2358 9582211

[B96] Willett J. A. Cao J. Dorris D. M. Johnson A. G. Ginnari L. A. Meitzen J. (2019). Electrophysiological properties of medium spiny neuron subtypes in the caudate-putamen of prepubertal male and female Drd1a-tdTomato line 6 BAC transgenic mice. eNeuro 6:ENEURO.0016-0019.2019. 10.1523/eneuro.0016-19.2019 30899778 PMC6426437

[B98] Wolf M. E. (2010). The bermuda triangle of cocaine-induced neuroadaptations. Trends Neurosci. 33, 391–398. 10.1016/j.tins.2010.06.003 20655604 PMC2935206

[B97] Wolf J. A. Moyer J. T. Lazarewicz M. T. Contreras D. Benoit-Marand M. O’Donnell P.. (2005). NMDA/AMPA ratio impacts state transitions and entrainment to oscillations in a computational model of the nucleus accumbens medium spiny projection neuron. J. Neurosci. 25, 9080–9095. 10.1523/jneurosci.2220-05.2005 16207867 PMC6725747

[B99] Yizhar O. Fenno L. E. Prigge M. Schneider F. Davidson T. J. O’Shea D. J.. (2011). Neocortical excitation/inhibition balance in information processing and social dysfunction. Nature 477, 171–178. 10.1038/nature10360 21796121 PMC4155501

[B100] Yu J. Yan Y. Li K. L. Wang Y. Huang Y. H. Urban N. N.. (2017). Nucleus accumbens feedforward inhibition circuit promotes cocaine self-administration. Proc. Natl. Acad. Sci. U S A 114, E8750–E8759. 10.1073/pnas.1707822114 28973852 PMC5642706

[B101] Zarrindast M.-R. Bahreini T. Adl M. (2002). Effect of imipramine on the expression and acquisition of morphine-induced conditioned place preference in mice. Pharmacol. Biochem. Behav. 73, 941–949. 10.1016/s0091-3057(02)00951-6 12213541

[B102] Zerlaut Y. Destexhe A. (2017). Enhanced responsiveness and low-level awareness in stochastic network states. Neuron 94, 1002–1009. 10.1016/j.neuron.2017.04.001 28595044

[B104] Zhang X. F. Hu X. T. White F. J. (1998). Whole-cell plasticity in cocaine withdrawal: reduced sodium currents in nucleus accumbens neurons. J. Neurosci. 18, 488–498. 10.1523/jneurosci.18-01-00488.1998 9412525 PMC6793427

[B103] Zhang W. Linden D. J. (2003). The other side of the engram: experience-driven changes in neuronal intrinsic excitability. Nat. Rev. Neurosci. 4, 885–900. 10.1038/nrn1248 14595400

[B105] Zhou F. W. Chen H. X. Roper S. N. (2009). Balance of inhibitory and excitatory synaptic activity is altered in fast-spiking interneurons in experimental cortical dysplasia. J. Neurophysiol. 102, 2514–2525. 10.1152/jn.00557.2009 19692507 PMC2775391

